# Acupuncture Relieves Stress-Induced Depressive Behavior by Reducing Oxidative Stress and Neuroapoptosis in Rats

**DOI:** 10.3389/fnbeh.2021.783056

**Published:** 2022-01-04

**Authors:** Wen-Jing Cheng, Peng Li, Wen-Ya Huang, Yang Huang, Wen-Jie Chen, Yi-Ping Chen, Jun-Liang Shen, Jian-Kun Chen, Na-Sha Long, Xian-Jun Meng

**Affiliations:** ^1^Department of Traditional Chinese Medicine, School of Medicine, Xiamen University, Xiamen, China; ^2^Shenzhen Research Institute of Xiamen University, Shenzhen, China; ^3^Yueyang Hospital of Integrated Traditional Chinese and Western Medicine, Shanghai University of Traditional Chinese Medicine, Shanghai, China; ^4^Institute of TCM-Related Comorbid Depression, Nanjing University of Chinese Medicine, Nanjing, China; ^5^Third Clinical College, Shanxi University of Traditional Chinese Medicine, Taiyuan, China

**Keywords:** depression, acupuncture, oxidative stress, Nrf2/HO-1 signaling pathway, neuronal apoptosis

## Abstract

Oxidative stress is closely related to the occurrence of depression. Acupuncture has been proved to be an effective method for treating depression. In order to explore the mechanism of the antidepressant effect of acupuncture, this study performed acupuncture prevention on chronic unpredictable mild stress (CUMS) depression model rats, and observed the effect of acupuncture on hippocampal oxidative stress and Nrf2 signaling pathway. Male SD rats were randomly divided into control group, CUMS group, acupuncture group, and fluoxetine group (*n* = 10/group). Fluoxetine, a common antidepressant, was used as a positive control drug in this study. In the fluoxetine group, rats were given fluoxetine (2.1 mg/kg) intragastrically once a day for 28 days. The acupoints of *Shangxing* (GV23) and *Fengfu* (GV16) were applied in acupuncture group, once every other day for 14 times in total. Behavioral tests and biological detections were used to evaluate the effects of the interventions and the changes of factors related to oxidative stress, Nrf2 pathway, and neuronal apoptosis. The results showed that both acupuncture and fluoxetine could increase sugar preference rate in SPT and decrease immobility time in FST in depression model rats. It also significantly decreased oxidative stress products such as ROS and H_2_O_2_, and elevated the protein and mRNA expressions of Nrf2 and HO-1. From Nissl’s staining, there were more abundant nerve cells in two intervention groups compared with CUMS group. Plus, acupuncture down-regulated the expression levels of Bax and caspase-3 and up-regulated the expression of Bcl-2. Our findings indicate that acupuncture improved depression-like behaviors of CUMS rats. And CUMS-induced depression-like behaviors in rats were related to oxidative stress and neuronal apoptosis in hippocampus. Acupuncture showed antidepressant effects in reducing oxidative stress products via regulating the Nrf2/HO-1 signaling pathway so that prevented neuronal apoptosis.

## Introduction

Depression is a mental illness associated with depressed mood, loss of interest, and energy fatigue (Zhou et al., 2019). Not only it causes serious damage to the patients but also imposes a huge economic burden on society due to its high morbidity rate and recurrence rate (Jun et al., 2014; Can et al., 2018). However, antidepressants were commonly used in clinic now, such as tricyclic antidepressants and serotonin reuptake inhibitors, which have a series of side effects and even no effect (Li K. et al., 2020). Therefore, the exploration of new treatments for depression is needed.

Studies have reported that the occurrence of depression is closely relevant to oxidative stress (Dantzer et al., 2008; Reiter et al., 2020), which subsequently leads to neuronal apoptosis (Khan et al., 2019). Such responses are, in a way, due to environmental stimuli which then change neuroprogression resulting in the evolution of depression-like behaviors (Berk et al., 2013). Some clinical reports have stated that oxidative stress is one of the main causes of the pathogenesis of depression (Ng et al., 2008), as indicated by elevated levels of oxidative stress markers found in the blood of patients with depression (Lindqvist et al., 2017). In addition, antidepressants are effective in reducing oxidative damage in depressive patients (Ng et al., 2008; Maes et al., 2011). What’s more, antioxidant subjects such as polyphenols exhibited antidepressant activity by modulating oxidative stress state in the brain of depression model animals (Chhillar and Dhingra, 2013).

Nuclear factor erythroid 2-like (Nrf2) is an intracellular transcription regulator, and Heme oxygenase-1 (HO-1) is one of its most important downstream regulatory products. The cascade reaction between the two is critical in the brain antioxidant system. Furthermore, the antioxidant function of the brain is linked to the pathophysiology of depression (Black et al., 2015). In a previous study, carvacrol ameliorated LPS-induced depression and anxiety-like behavior by regulating the neuroprotective properties of endogenous antioxidant protein Nrf2 (Naeem et al., 2021). Hence, Nrf2 might be considered as a potential pharmacological target for studying the mechanism of depression.

Acupuncture, as a traditional Chinese medical therapy, is often used as a complementary and alternative treatment for depression (Smith et al., 2018). Based on mountains of ancient books, we found that in Sun Simiao’s Thousand-Golden-Prescriptions (Bei Ji Qian Jin Yao Fang), thirteen ghost acupoints were often used to treat mental diseases, including depression (Si-yu et al., 2020). Although acupuncture has a certain effect in ameliorating depression, its molecular mechanism is not yet clear. For instance, previous researches found the reduction of the severity of depression in patients treated with acupuncture (Smith et al., 2018). Certainly, acupuncture is considered as an alternative treatment in primary care to significantly reduce depressive symptoms in the short to medium term (MacPherson et al., 2013). In our previous study, acupuncture improved the concentration of 5-HT in the hippocampus of CUMS-induced depressed model rats (Li et al., 2021). The hippocampus, as a key brain region belonging to the limbic system, is responsible for physical functions such as memory formation, cognitive ability, and emotion regulation (Slavich and Irwin, 2014). These observations underscore that it is indispensable to assess the rational utility of acupuncture in depression and to investigate the mechanisms involved. However, it is still unknown whether acupuncture could relieve neural injury via decreasing oxidative stress leading to apoptotic responses correlated with depressive behavior. Herein, we designed this experiment to explore whether acupuncture could suppress oxidative stress and alleviate neuronal apoptosis in the hippocampus of rats. Interestingly, our results suggest acupuncture is a potential preventive antidepressant and emphasize that oxidative stress and neuronal apoptosis are likely to be targets for the study of depression.

## Materials and Methods

### Animals

Male Sprague-Dawley (SD) rats of specific-pathogen-free (SPF) class weighting 130–150 g, were obtained from Shanghai Slake Laboratory Animal Co., Ltd. [Animal Certificate No. SCXK (Shanghai) 2017-0005]. Animals were kept in a room where the temperature (22 ± 2°C) and humidity (50 ± 10%) were controlled. Before the treatment, rats were adapted to the environment for 1 week. This experiment was conducted in accordance with ARRIVE’s guidelines. In addition, all operations on rats were confirmed with ethical regulations and the ethics committee of animal care of the Xiamen University, Xiamen, China (License No. XMULAC202110062).

### Chronic Unpredictable Mild Stress (CUMS) Induction Protocol

The rat model of depression was established by chronic unpredictable mild stress (CUMS) as described previously with a slight modification (Wang et al., 2017). In this study, 40 baseline-screened rats were randomly divided into 4 groups: control group (con, *n* = 10), CUMS group (cums, *n* = 10), acupuncture group (acu, *n* = 10), fluoxetine group (flu, *n* = 10). In addition to the control group, the other three groups were isolated in individual cages and received different stressors every day during the 28 days. Each stressor could not occur continuously. Our stressors included water deprivation (24 h), food deprivation (24 h), restraint stress (6 h), wet bedding (24 h), smell stimulation, day and night reversal (24 h), strobe stimulation (6 h), and crowding (6 h).

### Acupoints and Drug

**FIGURE 1:**
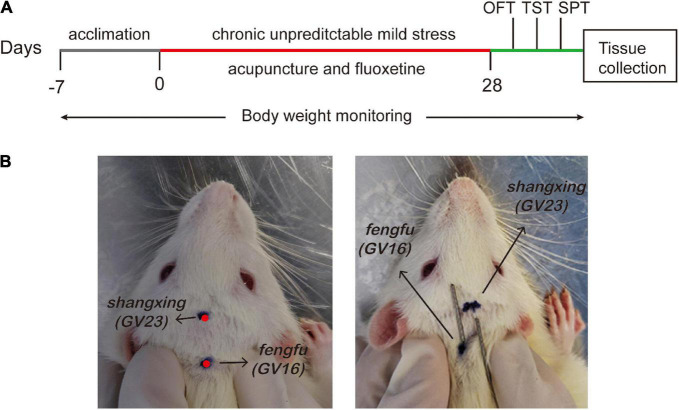
Schematic representation of experimental protocol. **(A)** Flow chart of experiment. **(B)** The location of acupoints (*Shangxing* GV23 and *Fengfu* GV16) in the rat.

In this experiment, acupuncture and fluoxetine should be performed 1 h before the CUMS protocol. According to the atlas in acupoints of experimental animals, two points were chosen (Fang, 1993; Figure 1): *Shangxing* (GV23) and *Fengfu* (GV16). GV23 is located in the middle of coronal line of rat anterior skull. GV16 locates at occipital posterior occipital ridge atlas joint. Both points were inserted as deep as 5–6 mm with sterilizing disposable stainless steel needles (0.25 mm diameter, HanYi, Changchun, China) for 20 min. An experimental rat head cap (no. ZL201922421814.3) was used to calm down the rats and thus ensure needles staying. Rats underwent acupuncture treatment every other day for 4 weeks. As a positive control, fluoxetine (2.1 mg/kg, 0.21 mg/ml, PHR1394-1G, Sigma-Aldrich) was diluted in double-distilled water and administered to rats daily for 4 weeks (Singh et al., 2004).

### Body Weight Monitoring

The body weight of each rat was measured at 9 am on day 0, 7, 14, 21, and 28 of the experiment, and the changes in body weight of each group were observed.

### Open Field Test (OFT)

According to previously described method (Luo et al., 2020), we performed open field test to determine locomotor activity, exploratory behavior, and depression in rodents. All rats were placed in an apparatus consisting of a dark rectangular case with a square floor (100 cm × 100 cm × 40 cm). The floor of the arena was divided into 4 × 4 equal squares. Then, each rat was placed individually in the center of the open-field apparatus and was allowed to freely explore the zone for 5 min. Finally, the total distance traveled (m) was recorded and analyzed by a behavioral recorder (Smart3.0). At the same time, the crossing numbers (defined as two forelimbs and one hindlimb in a square) were also recorded. After each trial, rat’s feces in the box were cleaned up and the open-field was thoroughly cleaned with 75% alcohol and dried.

### Sucrose Preference Test (SPT)

Sucrose preference test is used to evaluate anhedonia in rodents, which is a core symptom of depression (Forbes et al., 1996). The test was carried out following the previously described protocols with minor modifications (Yao et al., 2021). Before the formal test, rats were received two bottles of 1% sucrose solution for 24 h. Then change one bottle of sugar water to pure water for 24 h, when the position of the two bottles were exchanged every 12 h. Next, rats subjected to water deprivation and fasting for 12 h. Subsequently, each rat placed in a single cage, respectively, acquired one bottle of 1% sucrose solution and a bottle of pure water, of which weight calculated. After 12 h, the weight of sugar water and pure water were weighed again. Finally, the sucrose preference rate was calculated as follows: sucrose preference rate (%) = sucrose intake/(water intake + sucrose intake) × 100. The test was conducted at the end of the period of 4-week stress.

### Forced Swimming Test (FST)

Forced swimming test is often used to assess depression-like behavior in animals (Porsolt, 1993). After the sucrose preference test, the forced swimming test modified as described earlier was performed (Li S. Y. et al., 2020). All rats were subjected to forced swimming for 6 min in a transparent cylindrical tube (diameter 0.2 m, height 0.45 m, water depth 0.3 m, water temperature 22 ± 2°C) and then recorded its the time of immobility in water via a behavioral recorder (Smart3.0). To avoid the recording error of the instrument, the tap water in the cylinder should be changed before each rat was detected.

### Enzyme-Linked Immunosorbent Assay (ELISA)

Rats in each group were anesthetized with 0.3% pentobarbital (1 ml/100 g). Then hippocampus tissues were taken down, added with PBS homogenate, and centrifuged at 4°C for 20 min (12,000 RPM/min). Next, the supernatant was collected for detection. According to the instructions, the expression levels of ROS, MDA, SOD and GSH-Px in the supernatant were detected by ELISA kit (E-BC-K102, Elabscience). The absorbance was measured at 450 and 550 nm, and the content of each tissue index was calculated according to the standard curve.

### Western Bolt (WB)

The hippocampal tissue was ground and added to the lysate. After centrifugation, the protein concentration was determined by the BCA Protein Assay Kit (QB214754, Thermo Scientific), and then separated on the SDS-PAGE gel, and the separated protein was transferred to the PVDF membrane (K5NA8023B, Amersham). PVDF was immersed in a blocking solution containing 5% skimmed milk powder, incubated for 1 h at room temperature, and incubated with the primary antibody overnight at 4°C (anti-Nrf2, Proteintech, 16396, 1:1,000 dilution; HO-1, Proteintech, 27282, 1:1,000 dilution). The next day, the corresponding secondary antibody was added and incubated at 4°C for 1 h. After washing the membrane, the ECL developed color, exposed and developed in a dark room, and used ImageJ software for statistical analysis.

### Reverse Transcription-Polymerase Chain Reaction (RT-PCR)

Extract total RNA from hippocampus with 1 ml Trizol, the determined RNA concentration and purity. The Nrf2 and HO-1 primer sequences (Table 1) were identified from the consensus coding sequence (CCDS) database of the national center for biotechnology information (NCBI). Fluorescence quantitative PCR kit (RN03, Aidley Biotechnology Co., Ltd) and RT-PCR instrument were used to detect the mRNA expressions of each gene.

**TABLE 1:** Primer sequence.

Gene	Primer sequence (5′–3′)	Product length (bp)
Rat-β-actin-F	CTGGCTCCTAGCACCATGAA	180
Rat-β-actin -R	AAAACGCAGCTCAGTAACAGTC	
Rat-Nrf2-F	TCGCCCTGTGCCTCTTTG	148
Rat-Nrf2-R	CTAGGTGCCACTCGTCTCG	
Rat-HO-1-F	CGAAACAAGCAGAACCCA	192
Rat-HO-1-R	CACCAGCAGCTCAGGATG	

*F, forward; R, reverse; Nrf2, Nuclear factor erythroid 2-like; HO-1, Heme oxygenase-1.*

### Nissl’s Staining

Nissl’s staining is commonly used to observe the morphology and survival of hippocampal neurons in rats, as reported previously (Chang et al., 2019). Briefly, the brain was fixed by pouring into 4% paraformaldehyde via the vascular system through the rat’s heart. After the sample was embedded in paraffin and cut into 5 μm thick sections. Next Nissl Stain (toluidine blue method) was immersed in a temperature box at 55°C for 30 min. Finally, the hippocampal tissue lesion was observed by a fluorescence microscope (Leica DM4B, Germany) at a magnification of 400×.

### Immunohistochemical Staining (IHC)

Hippocampal tissue was paraffin-embedded, cut into 5 μm, and dehydrated by gradient ethanol. Then 3% H_2_O_2_ was used for incubation at room temperature for 30 min, and endogenous peroxidase was inactivated. 10% sheep serum was added for incubation at room temperature for 1 h, Next primary antibody anti-Bax (50599-2, Proteintech), anti-Bcl-2 (26593-1, Proteintech), and anti-caspase-3 (Ab184787, Abcam) were, respectively, added for incubation for 1 h, and secondary antibody was added for incubation for 1 h. After PBS rinsing, each tissue was added with 50 μl DAB chromogenic, re-dyed with hematoxylin, dehydrated, xylene permeated, and sealed. The positive expression of the protein was observed under a 100-fold and 400-fold microscope.

### Statistical Analysis

All data were input into the computer and processed with GraphPad Prism 8.0.2 Windows software. Normality and homogeneity of variance were tested first. For a pairwise comparison between the groups that met the normal conditions, one-way ANOVA was used. If the variance was not homogeneous, the Brown-Forsythe ANOVA test was selected and the statistical data were expressed as mean ± standard deviation (x ± s). The non-parametric rank-sum test was used when the normality was not satisfied. All results with a difference of *P* < 0.05 were statistically significant.

## Results

### Effect of Acupuncture on Depressive Behaviors in the Chronic Unpredictable Mild Stress-Induced Rats

**FIGURE 2:**
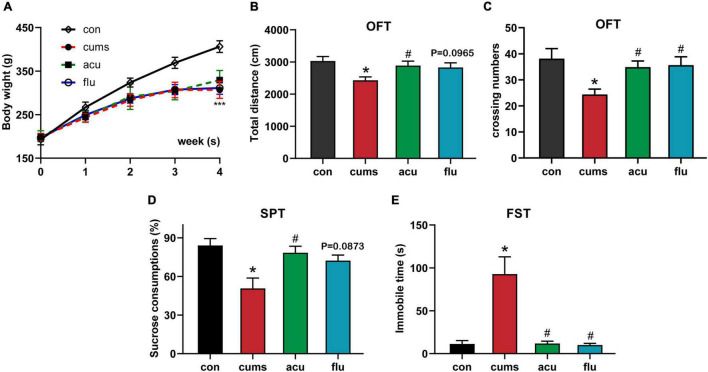
Acupuncture ameliorates depressive-behavior rats exposed to CUMS. **(A)** Acupuncture prevented the decreased the weight gain in CUMS rats. **(B,C)** Acupuncture or fluoxetine increases in total distance and crossing numbers of CUMS-exposed rats in the open field test. **(D)** Acupuncture prevented the decreased consumption of sucrose solution in CUMS rats. **(E)** Acupuncture or fluoxetine reversed the increases in immobility times of CUMS-exposed rats in the forced swim test. All values are presented as means ± SEM (*n* = 8), *P < 0.05, ***P < 0.001 compared to the control group, ^#^*P* < 0.05 compared to the CUMS group.

As shown in Figure 2, compared with control group, weight gain [*F*_(3_,_30_._82)_ = 62.55; *P* < 0.001], sucrose intake [*F*_(3_,_24_._99)_ = 6.162; *P* < 0.05] and total distance [*F*_(3_,_26_._77)_ = 3.827; *P* < 0.05] decreased, and immobility time [*F*_(3_,_7_._952)_ = 15.67; *P* < 0.05] increased in the model group, all of which were statistically significant. However, acupuncture significantly reduced CUMS-induced immobility time in FST, increased the distance traveled in OFT, and the sucrose preference rate in SPT (*P* < 0.05, *P* < 0.05, *P* < 0.05). These indicate that acupuncture can well alleviate depression-like behaviors caused by CUMS.

### Effect of Acupuncture on Oxidative Stress in the Hippocampus of Chronic Unpredictable Mild Stress-Induced Depression Rats

**FIGURE 3:**
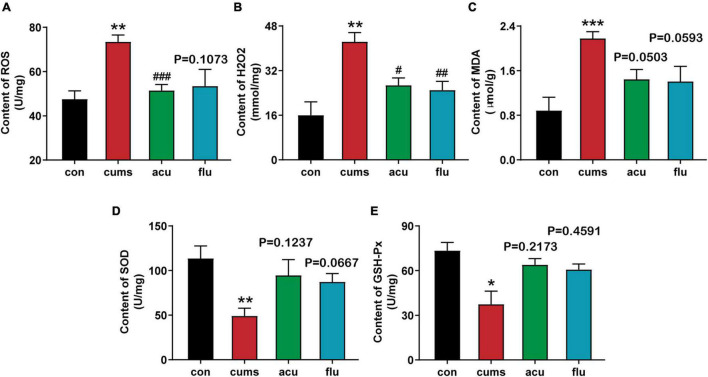
Acupuncture relieves the oxidative stress response in hippocampus of CUMS rats. **(A)** Acupuncture alleviated the increased levels of ROS within the hippocampal region caused by CUMS exposure. **(B)** Acupuncture and fluoxetine reduce oxidative stress product H_2_O_2_ in hippocampus of rats with depression. **(C–E)** Hippocampus level of MDA, SOD, GSH-Px. All values are presented as means ± SEM (*n* = 6), *P < 0.05, **P < 0.01, ***P < 0.001 compared to the control group. ^#^*P* < 0.05, ^##^*P* < 0.01, ^###^*P* < 0.001 compared to the CUMS group.

As shown in Figure 3, the activities of antioxidant enzymes, such as SOD and GSH-Px, were significantly decreased in the depression group compared with the non-stress control group (*P* < 0.01, *P* < 0.05). And compared with the unstressed control group, the levels of malondialdehyde (MDA) (*P* < 0.01) and hydrogen peroxide (H_2_O_2_) [*F*_(3_,_16_._15)_ = 9.419; *P* < 0.001] of oxidative stress products in the hippocampus were significantly increased after 4 weeks of exposure to CUMS. In addition, excessive production of reactive oxygen species (ROS), a sign of oxidative stress, under environmental stress can cause severe damage to the brain (Salim, 2017). The results showed that compared with the unstressed control group, ROS level in the hippocampus was significantly increased after 4 weeks of exposure to CUMS [*F*_(3_,_10_._85)_ = 6.038; *P* < 0.01]. In contrast, acupuncture could reduce the excessive accumulation of ROS in the hippocampus of rats (*P* < 0.001). From the above observation, we found that acupuncture prevented the above changes in oxidative stress caused by exposure to CUMS.

### Effect of Acupuncture on the Hippocampal Nuclear Factor Erythroid 2-Like Signaling Pathway in the Chronic Unpredictable Mild Stress-Induced Depression Rats

**FIGURE 4:**
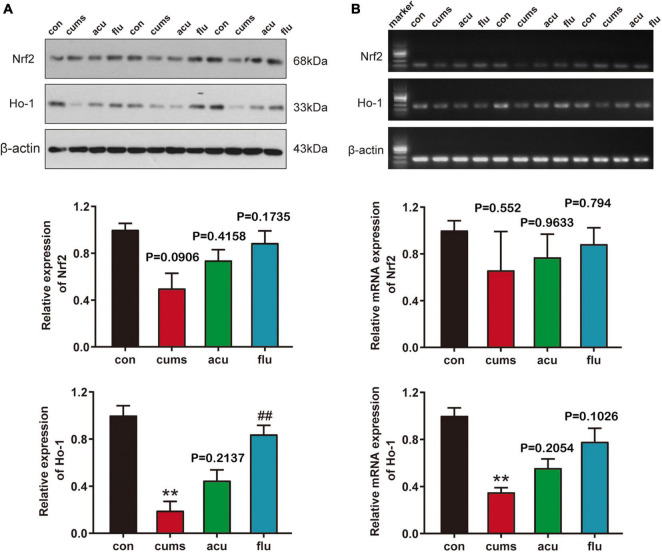
Acupuncture regulated the expression of Nrf2 signaling pathway in the CUMS-induce depressed rats of hippocampus. **(A)** The expression of Nrf2 and HO-1 protein in each group was detected by Western Blot. **(B)** The expression of Nrf2 and HO-1 mRNA in each group was detected by RT-PCR. All values are presented as means ± SEM (*n* = 3). con, control group; cums, CUMS group; acu, acupuncture group; flu, fluoxetine group, **P < 0.01 compared to the control group, ^##^*P* < 0.01 compared to the CUMS group.

The analysis revealed that the expression of HO-1 was significantly decreased as compared with the non-stressed control group after 4 weeks of CUMS exposure [*F*_(3_,_7_._884)_ = 19.67; *P* < 0.01]. Consistently, results from the RT-PCR analysis showed mRNA expressions of HO-1 significantly reduced as compared with control group [*F*_(3_,_5_._59)_ = 12.29; *P* < 0.01]. Interestingly, fluoxetine pretreatment elevated the protein expressions of HO-1 (*P* < 0.01). At the same time, the expressions of Nrf2 and HO-1 were increased via acupuncturing, although there was no statistical difference (*P* = 0.4158, *P* = 0.2137). It implied that acupuncture may improve the depression-like behaviors of rats by regulating the Nrf2 signal (Figure 4).

### Nissl Staining Detection of the Effect of Acupuncture on Hippocampal Neurons in the Chronic Unpredictable Mild Stress-Induced Depression Rats

**FIGURE 5:**
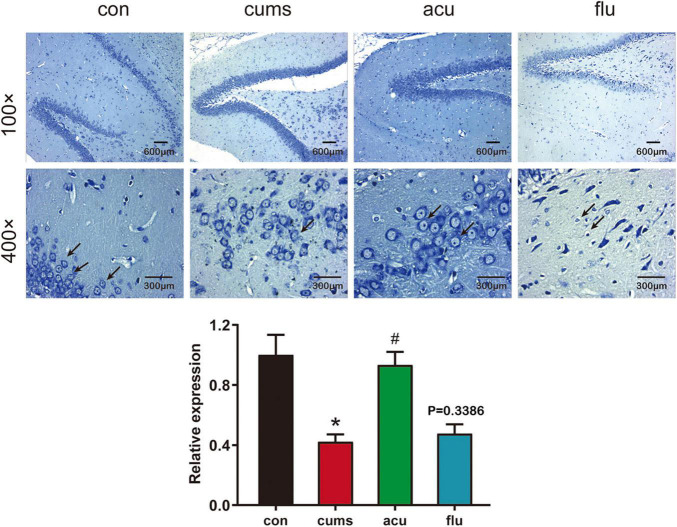
Acupuncture alleviated hippocampal neural injury in the CUMS-induced depressed rats. All values are presented as means ± SEM (*n* = 3). *P < 0.05 compared to the control group, ^#^*P* < 0.05 compared to the CUMS group.

The morphological and quantitative changes of hippocampal neurons were observed by Nissl staining (Figure 5). It could be found that the nerve cells of rats in control group were with high density, full cell body, and in order arrangement. At the same time, in the model group hippocampal neurons of rats were with large intercellular space and the cell layer was attenuated. The relative expression of pyramidal neurons was decreased after 4 weeks of CUMS exposure [*F*_(3_,_5_._008)_ = 11.29; *P* < 0.05]. Compared with the CUMS-induced group, acupuncture groups had increased numbers of normal Nissl bodies (*P* < 0.05).

### Effect of Acupuncture on Hippocampal Neuronal Apoptosis in the Chronic Unpredictable Mild Stress-Induced Depression Rats

**FIGURE 6:**
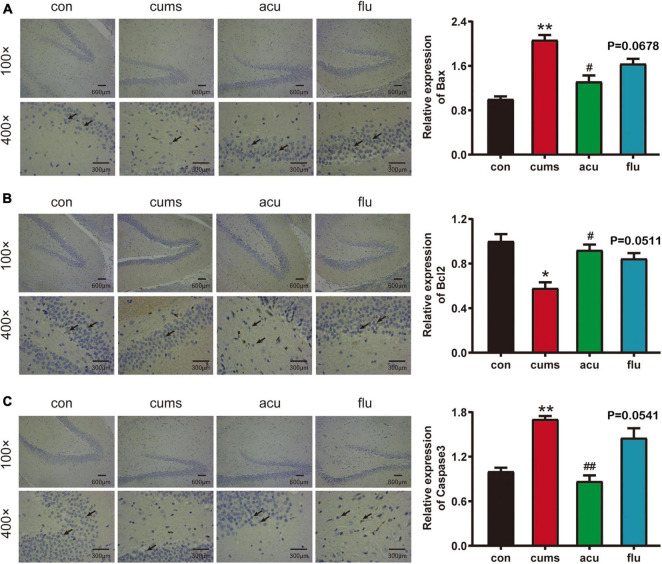
Acupuncture decreases the Hippocampal nerve apoptosis in the CUMS-induced depressed rats. **(A–C)** Are the relative expressions of Bax, Bcl-2 and caspase-3 in the hippocampus of rats in each group. All values are presented as means ± SEM (*n* = 3), *P < 0.05, **P < 0.01 compared to the control group, ^#^*P* < 0.05, ^##^*P* < 0.01 compared to the CUMS group.

The relative expression of Bax [*F*_(3_,_6_._716)_ = 26.01; *P* < 0.01] and caspase-3 (*P* < 0.01) were significantly increased in the hippocampus of CUMS-induce rats as compared with control group. On the contrary, compared with control group, the relative expression of Bcl-2 in the hippocampus of rats induced by CUMS was significantly decreased [*F*_(3_,_7_._618)_ = 10.99; *P* < 0.05]. However, the changes of apoptosis-related factors in the hippocampus of CUMS-induced rats of were significantly regulated in response to acupuncture pretreatment [*P* < 0.05, *P* < 0.05, *P* < 0.01]. These results suggest that the neuroprotective effect of acupuncture on apoptosis may help to restore the depression-like behavior of CUMS rats (Figure 6).

## Discussion

**FIGURE 7:**
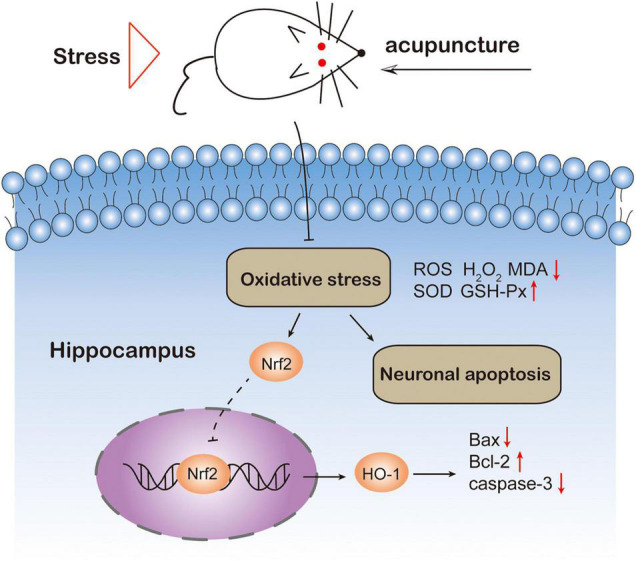
Schematic diagram of the mechanism of acupuncture preventing depression-like behavior in rats. Acupuncture *ShangXing* (GV23) and *Fengfu* (GV16) acupoints could suppress oxidative stress and neuronal apoptosis.

In this study, we evaluated the antidepressant effect of acupuncture in a CUMS-induced depression model in rats and explored its underlying mechanism (Figure 7). The results show that acupuncture can effectively prevent the occurrence of depression-like behaviors in CUMS rats. These helpful effects on the depressive behavior of CUMS rats is accompanied with the suppression of oxidative stress and the attenuation of hippocampal neuronal apoptosis. Taken together, our data suggest that the antioxidant effect of acupuncture on hippocampal neurons might be a potential therapy for depression.

To normalize the function of the body, acupuncture is used to stimulate specific sites in body to balance yin and yang in Traditional Chinese Medicine (TCM) theory (Wen et al., 2021). Based on this feature, some people in Southeast Asia apply acupuncture to treat mental illnesses such as depression (Seo et al., 2019). Indeed, according to a review on the effectiveness of acupuncture in treating depression, acupuncture could reduce the severity of depression (Smith et al., 2018). A clinical study reported that manual acupuncture enhanced therapeutic effectiveness and potentially reduced adverse effects caused by selective serotonin reuptake inhibitors (SSRIs) antidepressants (Zhao et al., 2019). Nevertheless, the molecular mechanism of the antidepressant effect of acupuncture needs to be explored. A latest report indicated that the specificity of acupoints is that they could drive a specific autonomic pathway: the vagal–adrenal axis (Liu et al., 2021). And in a previous study, acupuncture increased dendritic plasticity (Davila-Hernandez et al., 2018) and modulate BDNF expression (Davila-Hernandez et al., 2021) in hippocampus of rats with chronic social isolation. Besides, acupuncture altered hepatic lipid metabolism and inflammatory makers in the chronic restraint stress (CRS) induced depression model (Jung et al., 2021). Chronic unpredictable mild stress (CUMS) is currently the most common model of depression in that chronic external stressful life events are the crucial cause of depression (Willner, 2017). Previous studies have shown that acupuncture ameliorates inflammatory response in a rat model of CUMS (Lu et al., 2017). And electroacupuncture could reverse depression-like behavior and regulate astrocytes in the hippocampus in rats exposed to CUMS (Yao et al., 2021). In this study, a fluoxetine group was selected as the positive control group to evaluate the effect of acupuncture on CUMS rats. We found that acupuncture could prevent the depression-like behaviors in CUMS rats, showing a similar effect to the classic antidepressant fluoxetine. It is important to note that acupuncture may be considered in some patients who are unwilling to take antidepressants with side effects.

Reports have shown that oxidative stress plays an important role in the development of depression (Ng et al., 2008). Because the brain tissue has a high metabolic rate of oxidative phosphorylation, a high concentration of unsaturated fatty acid content, and a relatively low antioxidant capacity. Thus the brain is very sensitive to oxidative stress damage, and the resulting oxygen free radicals can damage neurons (Wang et al., 2016). Previous clinical studies have indicated that levels of several enzymes involved in the production of reactive oxygen species rose in depressed patients compared with controls that were not depressed (Scapagnini et al., 2012). Meanwhile, the expression of reactive oxygen species (ROS) and the lipid peroxidation marker malondialdehyde (MDA) were significantly increased in the CUMS-induced rats’ hippocampus (Abd El-Fattah et al., 2018). Indeed, our study also demonstrated that oxidative stress products in the hippocampus such as MDA and H_2_O_2_ were significantly enhanced in rats exposed to CUMS model compared to the non-stressed group. In addition, acupuncture pre-treatment successfully reversed CUMS-induced elevation of the ROS in the hippocampus, suggesting that the antidepressant effect of acupuncture might be ascribed to its antioxidant properties.

Previous studies have shown that the Nrf2/HO-1 signaling pathway plays an important role in resisting oxidative stress damage (Suzuki and Yamamoto, 2015). In resting state, Nrf2 and its inhibitor Kelch-like epichlorohydrin-related protein-1 (Keap1) are coupled in the cytoplasm and are in a relatively inhibited state (Sandberg et al., 2014). When cells are ischemic and hypoxic, Nrf2 dissociates from Keap1, and its nuclear translocates and binds to HO-1 which plays an antioxidant role in the process of cell stress (Bocci and Valacchi, 2015). In our present study, the results showed that chronic stimulation could significantly reduce the mRNA level of Nrf2 and HO-1. On the contrary, acupuncture could reverse these changes. The above results indicate that CUMS inhibited the Nrf2 signaling pathway. However, acupuncture pre-treatment increases the expression of antioxidant enzymes in the Nrf2 signaling pathway, thereby protecting the brain from CUMS-induced depression.

Depression could cause changes in the activity of enzymes related to oxidative stress. Compared with non-depressed controls, the levels of several enzymes involved in ROS production in depression patients increased (Scapagnini et al., 2012). Due to the peroxidation of cell membrane lipids, the increase of reactive oxygen species leads to the increase of MDA level. A clinical study revealed that the levels of MDA were higher in patients with depression than in healthy controls (Sarandol et al., 2007). Moreover, there was a significant reduction in some important ROS scavengers including superoxide dismutases (SOD), catalase (CAT), and glutathione peroxidase (GSH-Px) in rodent models of depression. Whereas treatments with some potential antidepressant drugs elevated the levels of these antioxidants to varying degree while also alleviated depression-like behavior (Hu et al., 2020; Sousa et al., 2021). Consequent to the increasing the marker of oxidative stress, the activation of pro-inflammatory correlation factors also contributes to the pathogenesis of depression (Bakunina et al., 2015). Taken together, these findings support a possible link between depression and oxidative stress injury. Studies also have shown that increased ROS activates various signal transduction pathways, leading to apoptosis and caspase cleavage (Wei et al., 2017; Yang et al., 2019). In addition, animal reports have indicated that the hippocampus is closely related to the occurrence of depression (Micheli et al., 2018). Hence, we used immunohistochemistry to detect the expression of apoptotic factors in the hippocampus of each group of rats. The results showed that, compared with the normal group, the expression of Bax in the hippocampus of the model group increased, and the expression of Bcl-2 decreased. However, acupuncture could relieve hippocampal cell apoptosis in CUMS rats, which is consistent with previous results (Guo et al., 2021).

In this study, the regulation of acupuncture intervention on oxidative stress and its influence on the Nrf2 signal pathway were preliminarily discussed. The present study provides new insights into the antidepressant effects of acupuncture underlying oxidative stress and the related signaling pathway. However, there are some limitations to be improved: considering the principle of animal welfare and reducing the number of experimental animals. In future experiments, in order to further study the intervention mechanism of acupuncture, we can choose to study the effect of antioxidant factors on apoptosis *in vivo*.

## Conclusion

All in all, the current research suggests that acupuncture is effective to treat the CUMS induced depressive rats, and the effect of acupuncture on depression-like behaviors induced by CUMS might be mediated by resisting oxidative stress response and anti-apoptosis.

## Data Availability Statement

The original contributions presented in the study are included in the article/supplementary material, further inquiries can be directed to the corresponding author.

## Ethics Statement

The animal study was reviewed and approved by the Ethics Committee of Animal Care of the Xiamen University, Xiamen, China (License No. XMULAC202110062).

## Author Contributions

X-JM: study design. W-JnC and PL: experimental filmmaker. W-JnC and W-JeC: manuscript writing. Y-PC and YH: statistical analysis. J-KC and J-LS: data analysis. N-SL and W-YH: manuscript review. All authors approved the final version of the article.

## Conflict of Interest

The authors declare that the research was conducted in the absence of any commercial or financial relationships that could be construed as a potential conflict of interest.

## Publisher’s Note

All claims expressed in this article are solely those of the authors and do not necessarily represent those of their affiliated organizations, or those of the publisher, the editors and the reviewers. Any product that may be evaluated in this article, or claim that may be made by its manufacturer, is not guaranteed or endorsed by the publisher.

## Abbreviations

CUMS: chronic unpredictable mild stress
Nrf2: Nuclear factor erythroid 2-like
HO-1: Heme oxygenase-1
OFT: open field test
SPT: sucrose preference test
FST: forced swimming test
SSRIs: selective serotonin reuptake inhibitors
ROS: reactive oxygen species
MDA: malondialdehyde
H_2_O_2_: hydrogen peroxide
SOD: superoxide dismutases
CAT: catalase
GSH-Px: glutathione peroxidase.
